# Triphala as a Topical Agent in Gynaecological Disorders: A Comprehensive Review of Its Pharmacological Actions and Applications

**DOI:** 10.7759/cureus.107796

**Published:** 2026-04-27

**Authors:** Anita, Latika Kundra, Poonam Verma, Nidhi Tahlan

**Affiliations:** 1 Department of Prasuti Tantra Evum Stri Roga (Obstetrics and Gynaecology), Gangasaran Sharma (GS) Ayurveda Medical College and Hospital, Pilkhuwa, IND; 2 Department of Panchakarma, All India Institute of Ayurveda, New Delhi, IND

**Keywords:** gynaecological disorders, integrative medicine, topical therapy, triphala, vulvovaginal infections

## Abstract

Gynaecological disorders, including abnormal vaginal discharge, vulvovaginal infections and chronic vulvovaginal inflammation, are common clinical problems for which locally acting therapies are particularly relevant. Triphala, a classical polyherbal formulation composed of *Terminalia chebula*, *Terminalia bellirica* and *Phyllanthus emblica*, has been traditionally used for mucosal and gynaecological conditions and is supported by experimental evidence suggesting antimicrobial, anti-inflammatory, antioxidant and wound-healing activities. This structured narrative review aimed at synthesising and critically evaluating the available evidence on the topical use of Triphala in gynaecological disorders, with an emphasis on pharmacological mechanisms, formulation strategies and reported clinical applications. Literature searches were conducted in PubMed, Scopus, Google Scholar and selected traditional medicine databases to identify English-language publications published between 2011 and December 2025. Experimental studies, clinical trials, and observational or clinical reports describing topical or locally applied Triphala formulations in gynaecological conditions or mechanistically relevant models were considered for qualitative synthesis. The reviewed literature suggests that Triphala-based topical formulations, including vaginal washes, intravaginal pessaries, tampon-based applications, medicated ghee preparations and semi-solid gels, may exhibit antimicrobial, anti-inflammatory, antioxidant and wound-healing properties at the vulvovaginal and cervical epithelium through modulation of inflammatory pathways, reduction of microbial burden, protection against oxidative stress and support of epithelial repair processes. However, the currently available evidence remains limited and heterogeneous, and further well-designed clinical investigations are required to clarify the therapeutic potential, safety and clinical applicability of Triphala as a topical botanical intervention in gynaecological practice.

## Introduction and background

Gynaecological disorders like abnormal vaginal discharge, vulvovaginal infection and chronic vulvar dermatoses are common clinical conditions that significantly affect the quality of life, sexual functioning and the functional status of reproductive and menopausal-aged women [1]. Local therapeutic modalities are also often used to treat these conditions, since their pathology is often confined to the vaginal or vulvar mucosa, where topical therapy can act directly on infection, inflammation and epithelial irritation. Vaginal discharge is known to cause symptomatic cases in 30% of women in India based on the epidemiological research studies carried out among women of childbearing age [2]. Bacterial vaginosis, vulvovaginal candidiasis and trichomoniasis infections were identified to be the most prevalent causes of this symptom complex and were found to account for a significant proportion of cases of vaginal discharge experienced in routine practice among women with symptomatic vaginal discharge [3]. Bacterial vaginosis, a condition involving disruption of the normal vaginal flora and proliferation of anaerobic organisms, is estimated to occur in 20%-30% of women of reproductive age worldwide, and it is also known to recur in half of the cases within six months of therapy [4]. These frequency trends indicate the need to identify safe, sustainable local treatment methods that can be adopted when necessary without causing resistance [5]. Another factor that plays a significant role in the general burden of vulvovaginal infections is recurring vulvovaginal candidiasis. Epidemiological surveys have indicated that the frequency of recurrent infections is high in the world, particularly among women of childbearing age [6]. Such epidemiological patterns highlight the essential role of topical therapy, which can potentially decrease recurrence rates and reduce the need for systemic and local antifungal treatment repeat exposure [7].

Besides infectious diseases, other causes of disease, including chronic inflammatory diseases of the vulva, are another group of conditions that require long-term local treatment. Persistent inflammatory disorders of the vulva, such as lichen sclerosus, usually necessitate lengthy treatment aimed at inhibiting inflammation and averting structural complications. For instance, topical corticosteroids with high potency have been suggested as mass-line therapy in case of chronic inflammatory lichen sclerosus of the vulva [8]. Though useful in the management of these disorders, continued administration of corticosteroids has to be closely monitored due to the local side effects. It has generated significant interest in research on topical modalities with good safety profiles as a form of adjunct or alternative therapies [9]. In traditional medical systems, symptom patterns similar to current signs of abnormal vaginal secretion, pruritus and topical inflammation have also been described [10]. These systems utilised regional therapeutic regimens for gynaecological disease management, vaginal irrigation, and tampon- and pessary-based delivery modalities designed to have a direct effect on the pathological focus [11]. Some of the botanical preparations formerly employed in such local applications include Triphala, a classical polyherbal preparation made of the fruits of *Terminalia chebula*, *Terminalia bellirica* and *Emblica officinalis* [12]. Triphala is an old folk medicine with general therapeutic benefits, and nowadays, it is in the scientific spotlight as a possible biomedical intervention.

It has been shown that Triphala is antimicrobial, antioxidant, anti-inflammatory and wound-healing through experimental and preclinical studies [13]. The anti-candidal activity has been observed in vitro, although other studies have shown that Triphala can modulate inflammatory pathways and promote epithelial healing [14]. The results provide a pharmacological foundation for exploring Triphala as a topical botanical formulation for gynaecological disorders typified by infection, inflammation and mucosal disturbance [15]. It is the syndromic heterogeneity of vaginitis, in addition to the high rate of recurrence of bacterial vaginosis and vulvovaginal candidiasis, that justifies the need to further explore standardised, pH-compatible Triphala-based vaginal preparations [16]. Further studies need to be conducted on well-articulated and clinically significant outcome measures, such as validated symptom severity scales, microbiological clearance and new microbiome-related outcome measures. To determine whether Triphala-based preparations can be properly integrated into evidence-based gynaecological care, a systematic assessment of local tolerability, long-term safety, optimisation of formulation parameters, dose, contact time and delivery vehicle, and analysis of interactions between Triphala-based preparations and currently used therapeutic strategies will be required.

Objective of the review

The objective of this review is to synthesise and critically evaluate the available preclinical and clinical evidence regarding the topical use of Triphala in gynaecological disorders, with a particular emphasis on pharmacological mechanisms, formulation approaches and reported clinical outcomes. Additionally, this review aims to identify key methodological limitations and evidence gaps in the existing literature to inform the design of future standardised formulations and prospective clinical studies assessing Triphala as a topical herbal therapy in gynaecological practice.

Methodology

The review was conducted using a structured narrative approach to synthesise available evidence on the topical use of Triphala in gynaecological conditions. A comprehensive literature search was performed in PubMed, Google Scholar, Scopus, Digital Helpline for Ayurveda Research Articles and the AYUSH Research Portal to identify both biomedical and ethnopharmacological studies. Peer-reviewed studies published in English between 2011 and December 2025 were considered. The search strategy included the terms “Triphala”, “topical application”, “vulvovaginitis”, “gynaecological disorders”, “vaginal therapy”, and “wound healing”, along with related terms describing local therapeutic interventions.

Titles and abstracts retrieved from the database search were screened for relevance, followed by full-text assessment according to predefined eligibility criteria. Studies were included if they evaluated Triphala formulations applied topically or locally in gynaecological conditions or in dermatological and wound-healing models considered mechanistically relevant to vulvovaginal or mucosal tissues. Preclinical experimental studies, observational studies, case series and clinical trials were eligible for inclusion.

From the selected studies, information on formulation type, concentration or dose, route of administration, treatment duration, reported outcomes and study limitations was extracted. Because of substantial heterogeneity in study design, experimental models and outcome measures, quantitative synthesis was not feasible. Therefore, findings were summarised using a qualitative narrative synthesis, and the extracted data were organised into summary tables to compare formulation approaches, therapeutic indications and observed effects. Potential methodological limitations and sources of bias were also considered during evidence interpretation.

## Review

Classical Ayurvedic background

Triphala (Triphala powder) is an Ayurvedic formula and a classical preparation of the dried fruit of *T. chebula* (Haritaki), *T. bellirica* (Vibhitaki), and *P. emblica* (Amalaki) [17]. Conventionally classified as a rejuvenation drug, Triphala has been suggested in pathologies of chronic inflammation, tissue degeneration and poor healing [18]. Classical preparations ascribe their medicinal value to effects equivalent to detoxification, astringency and antimicrobial activity, and are useful for skin disorders, skin wounds and gynaecological issues, such as abnormal vaginal discharge, vaginal infections, cervical lesions and uterine inflammation [19]. Such conventional signs are widely aligned with modern biomedical ideas of inflammatory control, antimicrobial effect and tissue healing stimulation [8].

Triphala is recognised for its rejuvenating and restorative properties. It supports overall vitality, longevity and tissue nourishment while enhancing skin health and delaying the effects of ageing through its antioxidant and detoxifying actions. It has demonstrated therapeutic potential for various skin disorders, including acne, psoriasis, eczema and wounds, due to its anti-inflammatory, antimicrobial, antipruritic and detoxifying effects. Additionally, it is used in the management of certain gynaecological conditions, administered as either internal or external applications due to its anti-inflammatory and antimicrobial activity, and its stimulation of tissue repair and healing.

Botanically and pharmacologically, Triphala is a mixture of deseeded, dried pericarps of mature fruits collected after the monsoon to achieve the highest concentration of hydrolysable tannins, a group of compounds associated with astringent and antimicrobial properties [20]. Conventional external preparations comprise aqueous decoctions to cleanse the wound bed, topical pastes for the inflammatory dermatoses and procedures to cleanse mucosal surfaces [16]. It has been shown that topical Triphala extracts can speed up the epithelialization process, build collagen, decrease oxidative stress and improve microbial clearance of infected dermal wound models [21]. These are experimentally verified properties offering a mechanistic foundation to the conventional application of Triphala decoction in localised gynaecological trials, specifically the conditions that involve the need to repair an epithelial area, decrease inflammatory exudation and the need for local antimicrobial activity [6].

Phytochemistry

Triphala possesses a high concentration of polyphenols and hydrolysable tannins, which, combined, elucidate an item of mechanical explanation behind its application in inflammatory and infectious gynaecological issues topically [22]. Gallic acid, ellagic acid, chebulinic acid and chebulagic acid are major bioactive constituents along with flavonoids and ascorbic acid [23]. Quantitative studies have demonstrated that Triphala contains about 34%-38% polyphenols and 32%-35% hydrolysable tannins by weight, which are well known as antimicrobials, anti-inflammatory, antioxidants, astringents and wound healers [18]. All of these phytochemicals contribute to the biological plausibility of Triphala as a topical botanical formulation in the treatment of conditions where mucosal inflammation, infection and lack of epithelial integrity may occur [14].

Pharmacologically, hydrolysable tannins have astringent and anti-exudative actions that could decrease excessive vaginal discharge, vascular oedema of the mucosa and congestion of the local vasculature [23]. Polyphenols and phenolic acids also assist in safeguarding the cervicovaginal epithelium by alleviating oxidative stress in addition to inhibiting chronic inflammatory signalling [24]. Experimental models of chebulinic and chebulagic acids have been shown to stimulate collagen production, induce epithelial repair and speed wound healing, all of which are applicable in cervical erosion and post-infectious mucosal lesions [16]. Moreover, flavonoid-based Triphala suppresses major pro-inflammatory cytokines, such as tumour necrosis factor-α and interleukin-6, mostly by regulating the nuclear factor-κB pathway [13]. Ascorbic acid also promotes the health of the mucosa by enhancing collagen synthesis and maintaining connective tissue structure [25].

These experimentally proven mechanisms provide a biomedical description that can be compared with the classical accounts of Triphala's local therapeutic action in gynaecological conditions [20]. Such phytochemical effects, when used topically, pertain to the treatment of ailments typified by abnormal vaginal secretions, inflammation of the mucosal lining, infection and slow healing, and hence justify further study on Triphala-based preparations in gynaecological studies and clinical trials.

Pharmacological mechanisms in cutaneous and mucosal models

Anti-inflammatory Property

Topical or local application of Triphala has been shown to have anti-inflammatory effects that can be measured in preclinical models, but these effects are mediated by selective inhibition of cyclooxygenase-derived inflammatory mediators and inhibition of nuclear transcription factor-kappa B (NF-κB)-induced cytokine pathways, as opposed to general inhibition of all eicosanoid pathways [22]. Topical Triphala formulations significantly decreased oedema formation in ethyl phenylpropiolate-induced rat ear oedema and had little effect on arachidonic acid-induced inflammation, indicating that the lipoxygenase pathway is not extensively involved [23]. Other studies have demonstrated a reduction in carrageenan-induced paw oedema and down-regulation of major pro-inflammatory mediators, such as tumour necrosis factor-α, interleukin-1β, interleukin-6, interleukin-17, monocyte chemoattractant protein-1, inducible nitric oxide synthase and cyclooxygenase-2, by the inhibition of NF-κB p65 activation [24]. The ability of Triphala to co-activate heme oxygenase-1 and endogenous antioxidant defences also adds weight to the possible application of Triphala as a non-steroidal, plant-based topical anti-inflammatory agent for cutaneous and mucosal inflammatory disorders.

Antioxidant Property

Triphala has been found to possess significant antioxidant activity in several studies [26]. In a study of animal models of bromobenzene-induced nephrotoxicity, Triphala was found to upregulate endogenous antioxidant enzymes like superoxide dismutase, glutathione S-transferase and glutathione peroxidase, which also decreased lipid peroxidation and tissue damage [20]. This suggests that Triphala increases the antioxidant potential of cells, which is relevant to all body tissues exposed to oxidative stress, such as mucosal epithelial cells. Further studies have confirmed that Triphala decreases stress-related oxidative damage in noise-stressed animals by reducing corticosterone levels and increasing antioxidant potential [27]. Although these models are associated with systemic oxidative stress, the mechanisms underlying the increase in endogenous antioxidant enzymes and the decrease in oxidative damage are relevant to epithelial tissues, where oxidative stress induces inflammation and disrupts epithelial barrier function. In vitro studies using human skin cells have shown that Triphala extract increases antioxidant enzyme gene expression, such as SOD2, and increases elastin and collagen type I production, in addition to reducing melanogenesis and hyperpigmentation [28]. It has been shown to protect against hydrogen peroxide-induced cellular damage and senescence, indicating a high potential for free radical-scavenging activity [18].

Wound-Healing Property

The wound-healing effects of Triphala in excision and incision wound models are consistent in preclinical evidence [29]. Topical use of a 10% Triphala ointment dramatically shortened wound contraction and wound closure, decreased bacterial load and elevated biochemical measures of tissue healing by assessing collagen, hexosamine, uronic acid and superoxide dismutase [26]. Such effects were coupled with the down-regulation of matrix metalloproteinases, which implies the presence of coordinated antibacterial, antioxidant, and pro-repair activities [16]. It has also been reported in clinical case reports to be successful in healing complex wounds such as diabetic foot ulcers using Triphala-based wound irrigation and topical applications, with slough and discharge reduction, formation of healthy granulation tissue and eventual wound healing [18]. Triphala decoction irrigation has been used in combination, leading to limb salvage in isolated cases of diabetic foot osteomyelitis [30]. Antimicrobial activity of Triphala decoction and alcoholic extracts against wound-related pathogens, including *Staphylococcus aureus*, *Escherichia coli*, Citrobacter species and Proteus species, has also been demonstrated, with stronger activity against Gram-positive organisms in vitro [31].

Antimicrobial Property

**Figure 1:**
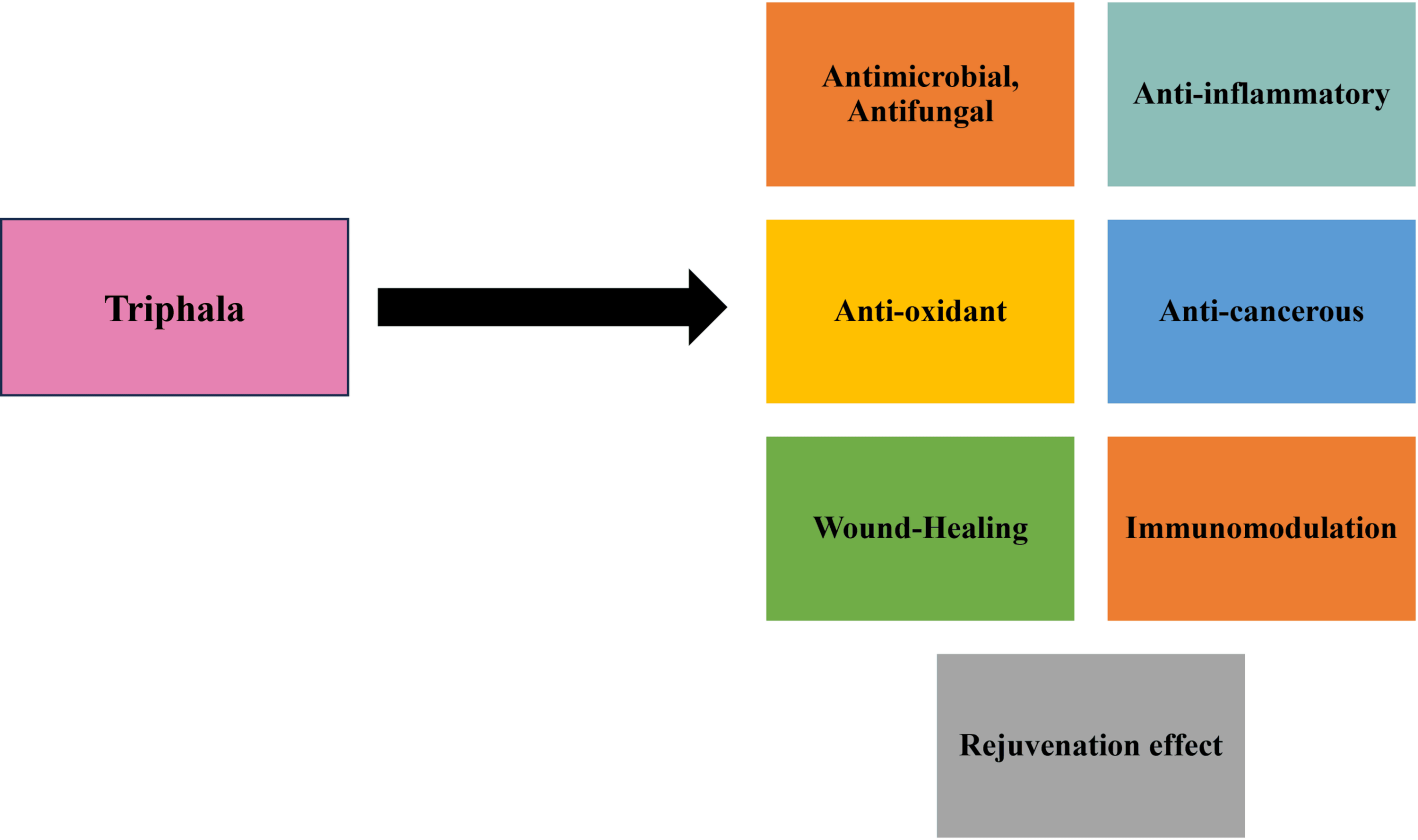
Pharmacological mechanisms of Triphala in gynaecological disorders Image credit: This is an original image created by the authors using Microsoft PowerPoint (Microsoft, Redmond, WA)

Triphala has been found to possess antimicrobial properties that are largely attributed to the presence of polyphenolic acids such as gallic acid, ellagic acid, chebulinic acid and chebulagic acid, particularly from *T. chebula*, along with flavonoids such as quercetin and luteolin [32]. These compounds exhibit synergistic antimicrobial activity through mechanisms including disruption of microbial cell walls, inhibition of enzymes, interference with microbial adhesion and inhibition of biofilm formation [28]. Triphala has been shown to demonstrate high antibacterial activity against *Enterococcus faecalis*, and in vitro studies have indicated superior antimicrobial efficacy compared with low concentrations of sodium hypochlorite when used as an endodontic irrigant, suggesting potential use as a less cytotoxic antimicrobial agent [17]. Other in vitro investigations have demonstrated inhibitory effects against oral and skin pathogens, including *Streptococcus mutans*, *S. aureus*, and *Actinobacillus actinomycetemcomitans*, with bacterial growth suppressed at defined minimum inhibitory concentrations [13]. Figure 1 provides a schematic summary of the principal pharmacological activities of Triphala in relation to its proposed use in the treatment of gynaecological disorders.

Topical Triphala formulations in gynaecological studies

The route of drug administration is significant because it determines therapeutic effectiveness, bioavailability, onset of action, duration of effect and the likelihood of systemic side effects [33]. Topical or local application of gynaecological treatment is of particular importance in disorders involving the vulva and vagina, as it allows localisation of active agents to the pathological site of disease [29]. Local delivery of drugs by vaginal route is particularly favourable because of the large surface, rich vascularity and the fact that water-soluble and lipophilic drugs of low molecular weight can be accommodated, allowing effective drug delivery locally [22]. Moreover, the first-pass hepatic metabolism of drugs administered via the vaginal route is bypassed, thereby minimising systemic exposure and toxicity associated with drug distribution, which is why this approach is attractive for the prevention and therapeutic control of recurrent genital infections [34].

**Figure 2:**
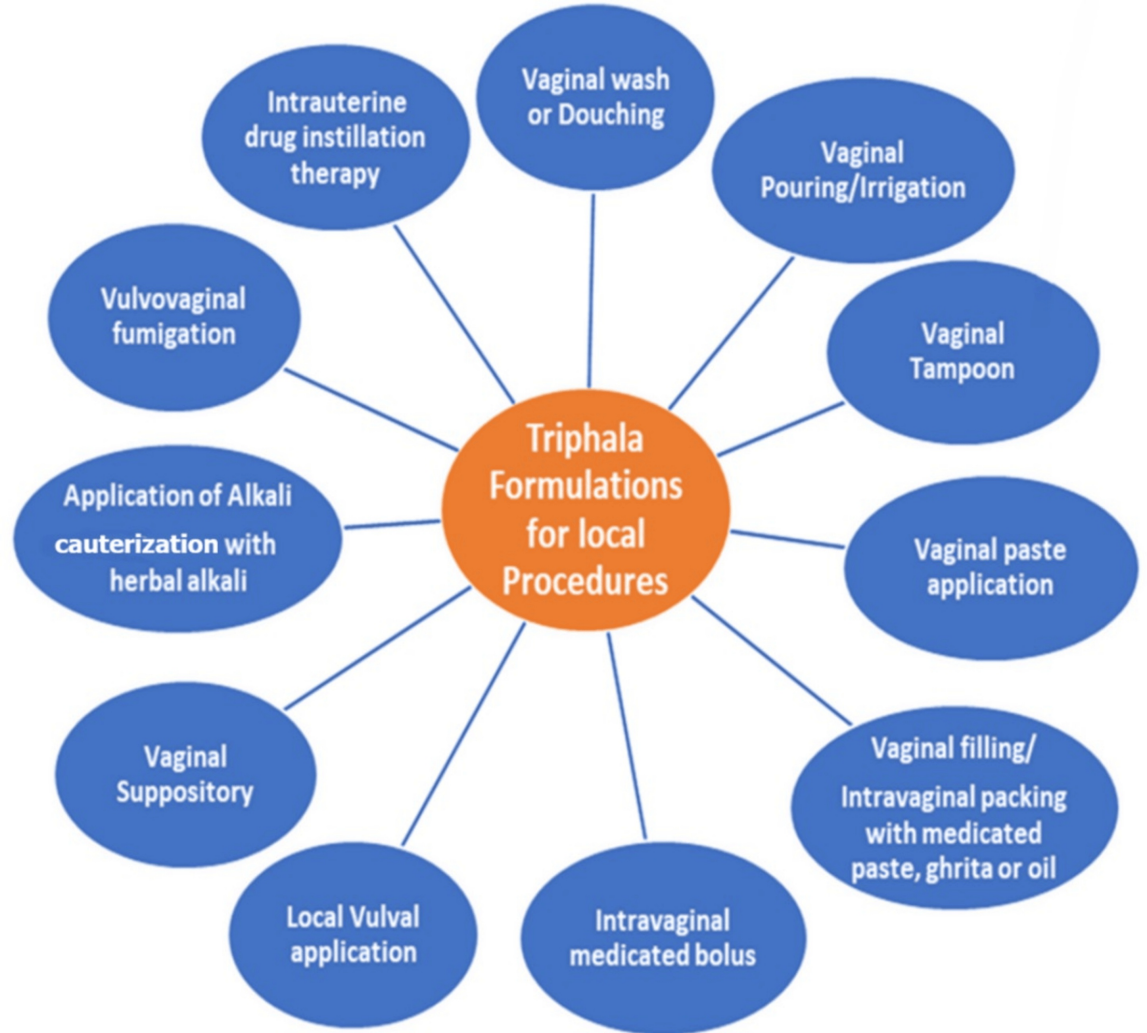
Topical Triphala formulations used in local gynaecological therapy Image credit: This is an original image created by the authors using Microsoft Word SmartArt (Microsoft, Redmond, WA)

In conventional gynaecology treatment, local therapeutic approaches have been employed to manage various vaginal and vulvar conditions using diverse formulation strategies [27]. In modern biomedical terminology, these include vaginal irrigation, drug delivery devices such as tampons, pessaries, and topical gel or ointment application [35]. Triphala has been incorporated into such local delivery systems in multiple gynaecological studies, including aqueous decoctions for vaginal washing, solid or semi-solid intravaginal formulations, and topical preparations applied to vulvovaginal tissues [24]. The primary objective of these formulations is to achieve high local concentrations of bioactive phytochemicals to exert antimicrobial, anti-inflammatory and wound-healing effects at the mucosal surface while minimising systemic exposure [30]. Figure 2 presents a schematic of commonly used Triphala-based topical preparations and their routes of local application in gynaecological practice.

Table 1 summarises commonly reported clinical contexts, formulation approaches and observed local effects of topical Triphala in gynaecological practice, synthesised from published preclinical and clinical literature.

**Table 1:** Clinical applications of topical Triphala in gynaecological practice Credit: The table was compiled by the authors based on data extracted and synthesised from [29,31,34-36]

Gynaecological condition	Topical formulation and route	Observed local effects	Typical duration of use	Reference
Post-procedural or post-partum perineal wounds	Aqueous Triphala decoction for local cleansing combined with lipid-based topical application	Reduction in local pain, oedema, tenderness and exudative discharge; support of wound healing and tissue comfort	Short-term application, typically within 1 week	[36]
Recurrent lower urinary and vaginal symptoms	Aqueous Triphala decoction is used as vaginal irrigation	Improvement in burning micturition, vaginal discharge and associated lower genital tract irritation	Repeated short treatment cycles	[34]
Vaginal infections associated with altered reproductive function	Vaginal irrigation with Triphala decoction, sometimes combined with systemic supportive measures	Resolution of vaginal infection and restoration of the local vaginal environment	Approximately 1 week	[29]
Vulvar pruritus and non-specific vulvovaginal irritation	Vaginal wash using Triphala decoction with adjunctive intravaginal lipid-based application.	Reduction in itching, discharge, odour and local discomfort; improvement in vaginal milieu	Short-term use over multiple cycles	[35]
Non-specific bacterial vaginitis	Triphala-based aqueous or polyherbal vaginal wash	Symptomatic relief, normalisation of vaginal pH and reduction in microbial burden	Short-term application, generally about 1 week	[31]

Table 2 presents a synthesised overview of commonly reported topical Triphala formulations, routes of administration and observed local effects in the management of vulvovaginal candidiasis.

**Table 2:** Topical Triphala formulations used in vulvovaginal candidiasis Credit: The table was compiled by the authors based on data extracted and synthesised from [29,34-36]

Clinical condition	Topical formulation and route	Observed local effects	Typical duration of use	Reference
Vulvovaginal candidiasis	Intravaginal herbal pessary containing Triphala	Reduction in vaginal discharge, pruritus and irritation with improvement in local symptoms	Approximately 1 week	[35]
Vulvovaginal candidiasis	Vaginal irrigation using Triphala-based decoction combined with an intravaginal pessary	Symptomatic relief with a reduction in fungal elements on local assessment	Short-term use, typically about 1 week	[29]
Vulvovaginal candidiasis	Vaginal wash with Triphala decoction combined with oil-based intravaginal application	Decrease in discharge and itching with improvement in vaginal pH	Short-term application	[34]
Vulvovaginal candidiasis	Local application of semi-solid herbal gel containing Triphala	Resolution of local symptoms with a reduction in fungal burden on microscopy	Repeated short treatment cycles	[35]
Vulvovaginal candidiasis	Vaginal irrigation using Triphala decoction with adjunctive lipid-based intravaginal application	Clinical improvement with a reduction in microbial growth on follow-up assessment	Short-duration application	[36]

**Figure 3:**
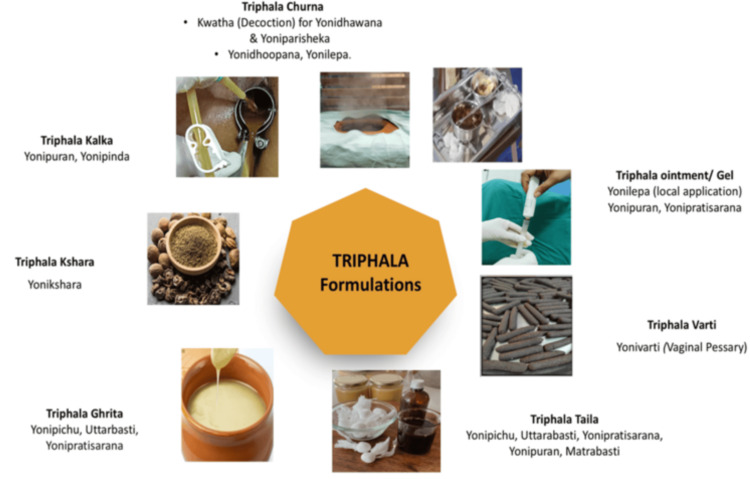
Local gynaecological applications of Triphala formulations Image credit: This is an original image created by the authors using Microsoft Word SmartArt (Microsoft, Redmond, WA)

Triphala formulations, including aqueous decoctions, intravaginal preparations such as pessaries and tampons, oil-based preparations, medicated ghee, and semi-solid gels, have been used to manage localised gynaecological conditions, particularly vaginal infections and inflammatory disorders of the mucosa [37]. These formulations enable the direct delivery of bioactive phytoconstituents to the mucosal surface, allowing high local drug concentrations to be achieved without hepatic first-pass metabolism [25]. Such localised delivery approaches are of interest in gynaecological practice due to efficient absorption through the vaginal route and minimal systemic exposure [12]. When applied locally, Triphala formulations have been reported to support cleansing, antimicrobial, anti-inflammatory and wound-healing activities at the vulvovaginal and cervical epithelium [26]. Vaginal irrigation with Triphala decoction has been described in the management of abnormal vaginal discharge and cervical pathology, while topical gels, ointments and tampon-based applications have been associated with healing of inflammatory lesions and surgical wounds [38]. Oil-based or lipid-rich formulations may enhance the mucosal penetration of lipophilic compounds, whereas ghee-based preparations have traditionally been employed to promote granulation tissue formation and local anti-inflammatory effects [34]. Intravaginal preparations of these pessaries have shown antifungal properties, particularly against *Candida albicans*, and may be responsible for the normalisation of vaginal pH through both cleansing and antimicrobial effects [39]. Beyond gynaecological application, extracts of Triphala have been reported to have antitumor effects in experimental models of ovarian, breast and cervical cancers, although the clinical applicability is still at the experimental level [40]. Figure 3 illustrates the various Triphala formulations and their gynaecological applications.

Mode of action of Triphala formulations when used as local therapy

The therapeutic effects of local applications of Triphala formulations are mediated by a range of physicochemical and biological activities relevant to vulvovaginal pathology [35]. In Ayurvedic pharmacology, it is stated that Triphala possesses astringent and drying properties (Kashaya Rasa and Ruksha Guna), which are considered to reduce excessive secretions, cleanse tissues and establish normal tissue balance. In biochemical and pharmacological terms, this is interpreted as a reduction of excessive mucosal secretions, modification of local inflammatory responses and enhancement of tissue repair processes. Such pharmacological activities are of particular relevance in clinical states of vaginal discharge, itching, burning, oedema and irritation of the mucosal surfaces [29]. The topical activity of Triphala is also mediated by molecular interactions of its constituents, which are rich in tannins, polyphenols, gallic acid and ellagic acid, and exert a number of pharmacological effects, including antibacterial and antifungal activity, suppression of pro-inflammatory cytokines, enhancement of antioxidant defenses and promotion of epithelial wound healing. The molecular structure of hydrolysable tannins may also restrict exudation and mucosal oedema through protein-precipitation activity, whereas polyphenols and phenolic acids may contribute to re-establishing epithelial integrity and immunomodulation in affected areas [31]. Accordingly, the Ayurvedic claims of reduced secretions and re-establishment of tissue balance in affected areas may also be interpreted in biochemical terms, with reference to the modulation of pro-inflammatory and oxidative pathways and microbial loads in affected areas of the body [35].

**Figure 4:**
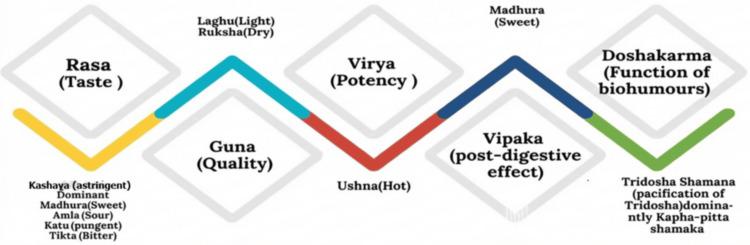
Traditional ayurvedic attributes of Triphala and their relevance to local therapeutic effects Image credit: This is an original image created by the authors using Microsoft Word SmartArt (Microsoft, Redmond, WA)

Triphala formulations may also exert beneficial effects in reference to the vaginal microenvironment, supporting vaginal pH and restricting the growth of pathogenic microorganisms in this area of the female reproductive system [34]. Reduction of inflammatory exudate and microbial loads in this area of the female reproductive system may also indirectly contribute to the re-establishment of a healthy vaginal microenvironment [29]. The antimicrobial, anti-inflammatory, antioxidant and wound-healing activities of Triphala formulations provide a biochemical basis to justify the localized gynaecological applications of this formulation, although this evidence is mostly limited to experimental and clinical investigations of localized applications of this formulation. Figure 4 illustrates the Ayurvedic pharmacodynamic profile of Triphala, including its Rasa, Guna, Virya, Vipaka and Doshakarma attributes.

**Figure 5:**
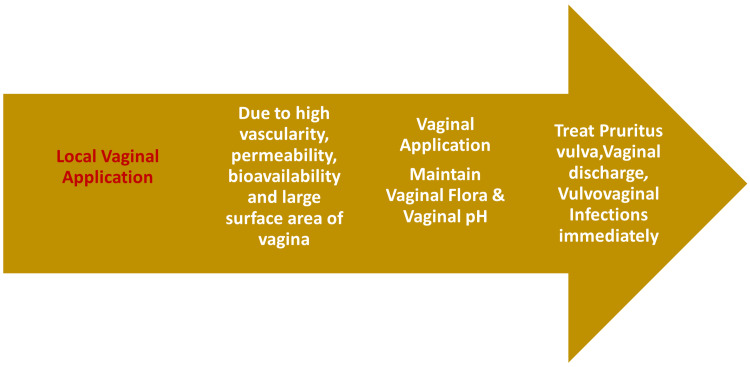
Local gynaecological effects of Triphala formulations on the vaginal microenvironment Image credit: This is an original image created by the authors using Microsoft PowerPoint (Microsoft, Redmond, WA)

Figure 5 illustrates the effect of vaginal application in vulvovaginal disorders and vaginal pH balance as key aspects of gynaecological health.

Safety and tolerability

The limited number of clinical and preclinical reports on topical preparations of Triphala suggests that, when properly prepared and applied, topically applied formulations of Triphala are generally well tolerated in gynaecological practice [35]. Observational reports of vaginal irrigation, sitz baths, intravaginal pessaries, tampon-based applications and topical gels or ointments have documented an absence of significant local adverse effects, such as burning sensation, mucosal irritation or discomfort [29]. These findings indicate a favourable local tolerability profile for various routes of administration with a special focus on the hygienic preparation, adequate filtration of the formulations and the hygienic administration of the products [34]. Topical administration of Triphala enables localised exposure of bioactive phytoconstituents to the vulvovaginal mucosa while minimising systemic uptake, thereby reducing the potential risk of systemic adverse events [31]. There are no serious adverse reactions, allergic reactions or discontinuation of therapy reported in clinical reports based on the use of Triphala as a short-term local therapy [30]. The lack of irritation associated with commonly used preparations, including aqueous decoctions, oil-based formulations, medicated ghee and semi-solid gels, further supports their suitability for mucosal application [32].

In vitro and experimental studies of Triphala extracts have also demonstrated a favourable safety profile at topical concentrations, with preservation of epithelial integrity and absence of overt cytotoxic effects in biological systems [36]. Collectively, these findings support the overall safety and local tolerability of Triphala-containing topical preparations in gynaecological practice, provided they are used following appropriate preparatory standards and within suitable clinical contexts.

Limitations and future recommendations

Topical application of Triphala is only available in limited clinical studies, case series and observational reports in the literature on the use of this compound in gynaecological disorders, thus limiting the overall extrapolation of results. The difference in type of formulation, mode of preparation, dose schedule and outcome measures among studies makes them less comparable. Besides this, most reports do not have long-term follow-up and standardised assessment instruments, and it is hard to make solid inferences about long-term efficacy and safety.

The future studies should aim at creating routine, pH-optimised Triphala-based vaginal preparations like gels, pessaries, foams and improved delivery systems. Randomised controlled trials with sufficiently large sample sizes should be developed to determine the efficacy and safety compared to conventional vaginal interventions. Dose response and contact time optimisation, microbiological and microbiome-based outcome measures, and long-term tolerability are the priority areas. Assessments of stability of formulations, mucosal compatibility and likelihood of interactions with intravaginal products (condoms and spermicides) will also be a necessity toward the support of evidence-based clinical practice.

## Conclusions

This review synthesises current preclinical and clinical evidence supporting the rationale for topical Triphala formulations in the management of localised gynaecological disorders, particularly conditions characterised by infection, inflammation, oxidative stress and impaired mucosal healing. The available literature indicates that Triphala exerts complementary antimicrobial, anti-inflammatory, antioxidant and wound-healing effects through well-described phytochemical mechanisms, including modulation of NF-κB-mediated inflammatory pathways, reduction of microbial burden, protection against oxidative injury and promotion of epithelial repair. Diverse topical delivery formats, such as vaginal washes, pessaries, tampon-based applications, medicated ghee and semi-solid gels, enable high local concentrations of bioactive constituents while minimising systemic exposure and associated adverse effects. Across reported studies, Triphala-based preparations demonstrate favourable local tolerability and safety when appropriately prepared and applied, supporting their suitability for short-term local use. The existing evidence base is limited by heterogeneity in formulations, small sample sizes and predominantly observational study designs. While the findings collectively support the pharmacological plausibility and potential clinical relevance of Triphala as a topical botanical therapy, its role remains investigational. Well-designed, standardised clinical trials are required to define optimal formulations, dosing strategies and long-term outcomes before Triphala can be integrated confidently into evidence-based gynaecological practice.
